# Development of a Real-Time Loop-Mediated Isothermal Amplification Assay for the Rapid Detection of Olea Europaea Geminivirus

**DOI:** 10.3390/plants11050660

**Published:** 2022-02-28

**Authors:** Sofia Bertacca, Andrea Giovanni Caruso, Daniela Trippa, Annalisa Marchese, Antonio Giovino, Slavica Matic, Emanuela Noris, Maria Isabel Font San Ambrosio, Ana Alfaro, Stefano Panno, Salvatore Davino

**Affiliations:** 1 Department of Agricultural, Food and Forest Sciences (SAAF), University of Palermo, Viale delle Scienze, 90128 Palermo, Italy; sofia.bertacca@unipa.it (S.B.); andreagiovanni.caruso@unipa.it (A.G.C.); danielaantonina.trippa@unipa.it (D.T.); annalisa.marchese@unipa.it (A.M.); 2 Research Centre for Plant Protection and Certification (CREA), Strada Statale, 113, 90011 Bagheria, Italy; antonio.giovino@crea.gov.it; 3 Institute for Sustainable Plant Protection, National Research Council (IPSP-CNR), Strada delle Cacce, 73, 10135 Turin, Italy; slavica.matic@ipsp.cnr.it (S.M.); emanuela.noris@ipsp.cnr.it (E.N.); 4 Instituto Agroforestal Mediteráneo, Universitat Politécnica de València (IAM-UPV), Camino de Vera, s/n, 46022 Valencia, Spain; mafonsa@upv.edu.es (M.I.F.S.A.); analfer1@etsia.upv.es (A.A.)

**Keywords:** LAMP, OEGV, *Geminiviridae*, olive viruses

## Abstract

A real-time loop-mediated isothermal amplification (LAMP) assay was developed for simple, rapid and efficient detection of the Olea europaea geminivirus (OEGV), a virus recently reported in different olive cultivation areas worldwide. A preliminary screening by end-point PCR for OEGV detection was conducted to ascertain the presence of OEGV in Sicily. A set of six real-time LAMP primers, targeting a 209-nucleotide sequence elapsing the region encoding the coat protein (AV1) gene of OEGV, was designed for specific OEGV detection. The specificity, sensitivity, and accuracy of the diagnostic assay were determined. The LAMP assay showed no cross-reactivity with other geminiviruses and was allowed to detect OEGV with a 10-fold higher sensitivity than conventional end-point PCR. To enhance the potential of the LAMP assay for field diagnosis, a simplified sample preparation procedure was set up and used to monitor OEGV spread in different olive cultivars in Sicily. As a result of this survey, we observed that 30 out of 70 cultivars analyzed were positive to OEGV, demonstrating a relatively high OEGV incidence. The real-time LAMP assay developed in this study is suitable for phytopathological laboratories with limited facilities and resources, as well as for direct OEGV detection in the field, representing a reliable method for rapid screening of olive plant material.

## 1. Introduction

The olive tree (*Olea europaea* L.) belonging to the *Oleaceae* family is the most widely cultivated species of the *Olea* genus. Olive, providing edible fruits and storable oil, has been cultivated in the Mediterranean area since prehistoric times [1], and is regarded as the most economically important fruit tree in the Mediterranean basin [2]. Olive cultivation has, over the centuries, played an important role in the economic development of rural areas in the Mediterranean region, providing noteworthy sources of income and employment opportunities for the population in rainfed agricultural territories [3]. Even today, after thousands of years, the countries in this area produce about 90% of the olive fruits [4], while the olive cultivated area covers about 10 million hectares worldwide. According to the latest data available on FAOSTAT, among Mediterranean countries, Spain ranked the major olive producer in 2020 (2,623,720 ha; 8.137.810 tons), followed by Italy (1,145,520 ha; 2,207,150 tons), Morocco (1,068,895 ha; 1,409,266 tons), Greece (906,020 ha; 2,790,442 tons), and Turkey (887,077 ha; 1,316,626 tons). According to ISTAT data [5], among the Southern Italian regions, Sicily (161,661 ha; 255,798 tons) played a significant role in olive and olive oil production, industry and export in 2021, being the third largest producer after Apulia (379,960 ha; 708,400 tons) and Calabria (184,410 ha; 680,275 tons). In Sicily, the olive crop has been cultivated since ancient times, and it is characterized by many ancient landraces/cultivars of high organoleptic quality [6]; its germplasm is distinguished by a wide genetic diversity, possibly related to its past domestication and spread and to some reproductive biological peculiarities such as self-incompatibility [7]. The production of olive oil in Sicily is based mainly on the autochthonous cultivars (cvs) ‘Biancolilla’, ‘Cerasuola’, ‘Moresca’, ‘Nocellara del Belice’, ‘Nocellara Etnea’, ‘Ogliarola Messinese’, ‘Santagatese’ and ‘Tonda Iblea’ [8]. The table olive industry is also appreciable, accounting to 10% of the total production of this region [5], mainly relying on the cv. ‘Nocellara del Belice’ and, to a minor extent, ‘Nocellara Etnea’, ‘Ogliarola Messinese’, and ‘Moresca’, produce large-sized fruits of high commercial value [8]. The current tendency in olive tree cultivation is moving towards the use of local cvs for high quality oil production (such as DOP—protected designation of origin), which is typical of specific geographic areas. For this reason, the local administration currently supports studies and activities aimed at the characterization and recovery of local and ancient cvs, in order to establish germplasm collections that limit genetic erosion [9]. However, a large number of diseases and disorders affect this crop, mostly caused by fungi, such as *Arthrinium phaeospermum*, *Phoma cladoniicola* and *Ulocladium consortiale*, recently discovered as new olive pathogens in Italy [10], but also by systemic pathogens including bacteria and viruses, which provoke significant yield losses. Indeed, in the last decade, olive production has suffered an enormous decline due to the emergence of biotic agents that have significantly undermined the Mediterranean economy related to olive and the olive oil industry; a dramatic example being the *Xylella fastidiosa* epidemic in 2013, which decimated olive trees in Apulia [11] and created huge losses for the local olive economy and oil production outputs, posing critical challenges for its management, as well as dramatic changes in the landscape [12,13]. Furthermore, the vegetative propagation of olive trees using cuttings of semi-wood has contributed over the years to the spread of systemic-pathogens, particularly viruses [14]. Nevertheless, despite the difficulty of associating specific symptoms to a particular virus, many viruses are easily transmitted through infected propagation material [15], and many olive infecting viruses are symptomless. Therefore, it is essential to better elucidate the evolutionary aspects of latent viruses in olive crops. In the last year, thanks also to the application of new technologies such as high-throughput sequencing (HTS), a new geminivirus called Olea europaea geminivirus (OEGV) has been identified in the olive tree [16], but its spread and pathogenicity remain puzzling. Since its first identification in Apulia [16] in the “Ogliarola” and “Leccino” cvs, OEGV was reported in California and Texas [17], Portugal [15], and Spain [18]. OEGV is classified as a putative member within the *Geminiviridae* family [16], currently including 14 genera [19] and few other still unassigned geminiviruses [20]. The evolutionary relationship of OEGV with other geminiviruses indicated that OEGV has distinctive genome features, possibly representing a new genus [15,16,17]. OEGV is characterized by a bipartite genome containing DNA-A and DNA-B. DNA-A (2775 nucleotides, nts) includes four ORFs, three in the complementary-sense encoding the replication-associated protein Rep (AC1), the transcriptional activator protein TrAP (AC2), the replication enhancer protein Ren (AC3) and one in the virion-sense, (AV1), encoding the coat protein (CP). DNA-B (2763 nts) includes two ORFs, BC1 in the complementary sense, with an unknown function and lacking known conserved domains typical of geminiviral proteins, and BV1 on the virion sense, possibly encoding the movement protein (MP). In bipartite geminiviruses, AC4/C4 protein is a symptom determinant involved in cell-cycle control, and interacts with CP and/or MP in the replicated genome transport from nucleus to cytoplasm and from cell-to-cell [15]. Curiously, no genes encoding AC4/C4 were found on the OEGV genome. In addition, the two DNA molecules present a common region (CR) of 403 nt that contains the TATA box and four replication-associated iterons with a unique arrangement compared to other geminiviruses [15,16,17]. In a recent survey, Alabi and co-workers [17] detected OEGV-positive olive trees originating from different locations, advancing the concept of a possible worldwide spread of this virus, likely due to the inadvertent movement of germplasms from clonally propagated infected but asymptomatic olive trees. As a matter of fact, OEGV does not appear to be clearly associated to any symptom in olive; moreover, a high degree of sequence conservation has been identified [18].

In this study, we aimed to investigate the presence of OEGV in Sicily and to develop a rapid detection protocol based on the LAMP methodology. In addition, an on-site olive sample homogenization procedure was developed replacing canonical DNA extraction methods, which is useful in evaluating the suitability of the LAMP assay for on-site OEGV testing.

## 2. Materials and Methods

### 2.1. Plant Material Collection

To investigate the presence of OEGV in Sicily, different surveys were carried out during spring 2021, focusing in particular on two olive producing sites in the Agrigento province (Sicily, Italy). The olive tree samples were randomly collected according to the hierarchical sampling scheme [21], with minor adaptations to olive plants. All samples were geo-referenced with the Planthology mobile application [22], collected from a total of 80 olive trees of 10 different cvs (40 trees randomly sampled for each site). Each sample consisted of 8 branches per plant (two for each plant cardinal point); samples were stored at 4 °C and processed within the next 24 h for subsequent molecular analyses.

### 2.2. DNA Extraction and Sample Preparation

Total DNA was extracted using the DNA extraction GenUP^TM^ Plant DNA kit (Biotechrabbit GmbH, Berlin, Germany), following manufacturer’s instructions with slight modifications. In brief, 3 g of tissue were homogenized in an extraction bag (BIOREBA, Reinach, Switzerland) using the HOMEX 6 homogenizer (BIOREBA, Reinach, Switzerland), with 3 mL extraction buffer (1.3 g sodium sulphite anhydrous, 20 g polyvinylpyrrolidone MW 24–40,000, 2 g chicken egg chicken albumin Grade II, 20 g Tween-20 in one L of distilled water, pH 7.4). Aliquots of 400 μL of the extract were added to the same volume of lysis buffer. The eluted DNA was resuspended in 100 μL RNase-free water; following two measurements with a UV–Vis NanoDrop 1000 spectrophotometer (Thermo Fisher Scientific, Waltham, MA, USA), samples were adjusted to approximately 50 ng/μL and stored at −20 °C.

### 2.3. Preliminary Screening of OEGV by End-Point PCR

The end-point PCR was conducted using the primer pair A2for/A4rev [16], amplifying an 831 bp fragment within the AV1 gene. PCR was performed in a final volume of 25 μL, containing 1 μL of total DNA extract, 20 mM Tris-HCl (pH 8.4), 50 mM KCl, 3 mM MgCl_2_, 0.4 mM dNTPs, 1 μM each primer, and 2 U Taq DNA polymerase (Thermo Fisher Scientific, Waltham, MA, USA) and RNase-free water to reach the final volume. Healthy olive plant DNA and water were used as control samples. The PCR was performed in a MultiGene OptiMax thermal cycler (Labnet International Inc., Edison, NJ, USA) with the following conditions: 95 °C for 5 min; 40 cycles of 95 °C for 30 s, 64 °C for 45 s, and 72 °C for 1 min; a final elongation at 72 °C for 10 min. PCR products were electrophoresed on 1.5% agarose gel, stained with SYBR^TM^ Safe (Thermo Fisher Scientific, Waltham, MA, USA) and visualized by UV light.

### 2.4. LAMP Primers Design

The OEGV DNA-A complete sequence (GenBank Acc. No. MW316657) was used to design LAMP primers by the PrimerExplorer version 5 software (http://primerexplorer.jp/lampv5e/, accessed on 5 July 2021), choosing a 540-bp nucleotide sequence elapsing region within the AV1 gene. A set of six primers were selected, including two outer primers (forward and backward outer primer, F3 and B3, respectively), two inner primers (forward and backward inner primer, FIP and BIP, respectively), and two loop primers (forward and backward loop primer, LF and LB, respectively). The specificity of the primer set was tested in silico using the Nucleotide-BLAST algorithm (https://www.ncbi.nlm.nih.gov, accessed on 5 July 2021) available at the National Centre for Biotechnology Information (NCBI), in order to evaluate possible cross reactions with other viruses. This set of primers was also tested against the full genomic sequences of other geminiviruses reported in Italy using the Vector NTI Advance 11.5 software (Invitrogen, Carlsbad, CA, USA), in order to verify their affinity. The list included Tomato leaf curl New Delhi virus (ToLCNDV) (DNA-A: GenBank Acc. No. MK732932 and DNA-B: MK732933), Tomato yellow leaf curl Sardinia virus (TYLCSV) (GenBank Acc. No. GU951759), Tomato yellow leaf curl virus (TYLCV) (GenBank Acc. No. X15656), TYLCV-IL23 (GenBank Acc. No. MF405078), and TYLCV isolate 8-4/2004 (GenBank Acc. No. DQ144621).

### 2.5. OEGV Real-Time LAMP Assay Optimization

The real-time LAMP assay was performed in a 12 μL reaction mixture containing 1.6 mM each of FIP-OEGV and BIP-OEGV, 0.2 mM each of F3-OEGV and B3-OEGV, 0.4 mM each of forward loop primer (LF-OEGV) and backward loop primer (LB-OEGV), 6.25 μL WarmStart LAMP 2X Mastermix (New England Biolabs, Beverly, MA, USA), and 0.25 μL of LAMP Fluorescent dye (New England Biolabs, Beverly, MA, USA), 1 μL of total DNA as template and nuclease-free H_2_O was added to reach the final volume. DNA extracted from ten samples previously analyzed by end-point PCR was used in the real-time LAMP assay, including a positive control (PC) and a healthy olive plant DNA as negative control (NC). Each sample was analyzed twice. The LAMP assay was conducted at 65 °C (according to manufacturer’s instructions) for 60 min and fluorescence was acquired every 60 s, using a Rotor-Gene Q2plex HRM Platform Thermal Cycler (Qiagen, Hilden, Germany). A melting curve was calculated to record the fluorescence using the following protocol: 95 °C for 1 min, 40 °C for 1 min, 70 °C for 1 min and an increase of temperature at 0.5 °C/s up to 95 °C. During the amplification, the fluorescence data were obtained in the 6-carboxyfluorescein (FAM) channel (excitation at 450–495 nm and detection at 510–527 nm). The relative fluorescence units (RFU) threshold value was used, and the threshold time (Tt) was calculated as the time at which fluorescence was equal to the threshold value.

### 2.6. Features of Real-Time LAMP Assay: Sensitivity and Comparison to Conventional PCR, Reaction Time and Specificity

To set up the conditions of the LAMP assay, an amplicon obtained by subjecting an OEGV-positive sample to end-point PCR (see above) was purified from agarose gel using an UltraClean™ 15 DNA purification kit (MO-BIO Laboratories, Carlsbad, CA, USA), following manufacturer’s instructions. The purified DNA (named pcr-DNA) was quantified using a UV–Vis NanoDrop 1000 spectrophotometer (Thermo Fisher Scientific, Waltham, MA, USA). The number of copies was determined as follows: [Number of copies = (amount of DNA in nanograms × 6.022 × 10^23^)/(length of DNA template in bp × 1 × 10^9^ × 650)]. To determine the OEGV real-time LAMP optimal reaction time and sensitivity, ten-fold serial dilutions of the sample (named pcr-DNA) were used as a template for both real-time LAMP assay and end-point PCR. Moreover, to evaluate the specificity of the LAMP assay and to assess potential non-specific cross reactions with other geminiviruses, a LAMP assay was conducted with two OEGV-positive samples together with DNA extracts from other geminiviruses unrelated to OEGV; specifically, ToLCNDV (Acc. No. MK732932) [23], TYLCSV (Acc. No. GU951759) [24], TYLCV (Acc. No. DQ144621) [25], TYLCV-IS76 [26]. Each sample was analyzed in duplicate in two independent real-time LAMP assays. In each run, total DNA from a healthy olive plant (NC) was included. The assay was conducted as above described, including the melting curve steps.

### 2.7. Set up of a Rapid Sample Preparation Method Suitable for the Real-Time LAMP Assay

To set up a simple and inexpensive sample preparation procedure, a method that avoided DNA extraction named “membrane spot crude extract” was used. For this, 1.5 g of vegetable tissue was placed in an extraction bag and homogenized with 3 mL of extraction buffer (see above). Five μL of this extract was spotted on a 1 cm^2^ Hybond^®^-N+ hybridization membrane (GE Healthcare, Chicago, IL, USA), dried at room temperature for 5 min, and placed in a 2 mL tube containing 0.5 mL of glycine buffer (0.1 M Glycine, 0.05 M NaCl, 1 mM EDTA). After 20-s vortexing, samples were heated at 95 °C for 10 min and 3 μL of the extract were used for the LAMP assay. Ten samples previously analyzed by end-point PCR were used in the real-time LAMP assay, including a positive control (PC) and a healthy olive plant DNA as negative control (NC).

### 2.8. Spread of OEGV in Different Cultivars

During autumn 2021, in different Sicilian areas, a second sampling was carried out to evaluate the OEGV spread in Sicily, this time sampling 10–15-year-old olive trees, belonging to 70 different cvs. A total of 560 samples were collected. For each cv, eight different trees were sampled and grouped, obtaining a total of 70 different batches. Sampling and geo-referencing were as described above. In this case, samples were prepared with the “membrane spot crude extract” method and subjected to real-time LAMP assays in a 12 μL final volume as described above. In the case of positive sample batches, they were re-sampled and analyzed individually to determine the effective number of positive plants for each cultivar.

## 3. Results

### 3.1. OEGV Detection by End-Point PCR

To ascertain the presence of OEGV in Sicily, a total of 80 samples representing 10 different cvs collected from two olive production sites in the Agrigento province were analyzed by end-point PCR. Overall, 44 of them were found to be positive to OEGV, demonstrating the presence of OEGV in Sicily also. However, OEGV was not equally distributed among the cvs tested, and some cvs tested negative for this virus, at least using the primer set mentioned in this manuscript (Table 1).

### 3.2. OEGV Real-Time LAMP Primer Design

**Figure 1:**
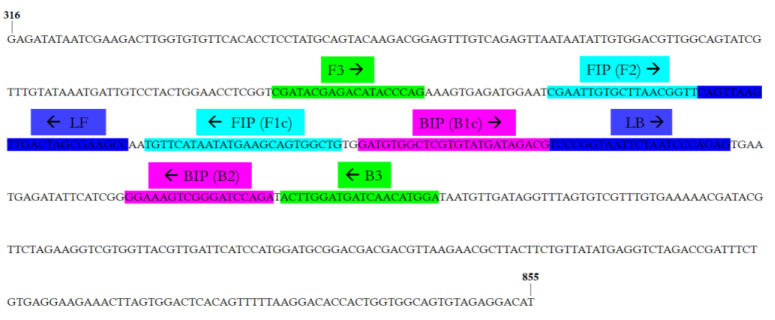
Location of loop-mediated isothermal amplification (LAMP) primer sets designed on the AV1 coding region of OEGV. F3 and B3 are shown in green, FIP (F1c-F2) in blue, BIP (B1c-B2) in pink, and the two loop primers LF and LB in brown. FIP is a hybrid primer consisting of the F1c and the F2 sequences, while BIP is a hybrid primer consisting of the B1c and B2 sequences. The arrows indicate the extension direction. The numbers at the beginning and end of the sequence represent the genomic position of the first and last nucleotide in the selected sequence (GenBank Acc. No. MW316657).

A real-time LAMP assay for the rapid detection of OEGV was developed using a set of six primers designed on the OEGV-AV1 coding region. The sequences and binding sites of the primers are reported in Table 2 and Figure 1, respectively.

Both the in silico analysis of LAMP primers using Nucleotide-BLAST algorithm and the hybridisation analysis against other geminiviruses performed with the Vector NTI 11.5 program allowed for the exclusion of relevant matches with other organisms and, more specifically, with geminiviruses known to be present in Sicily.

### 3.3. OEGV Real-Time LAMP Assay Optimization

**Figure 2:**
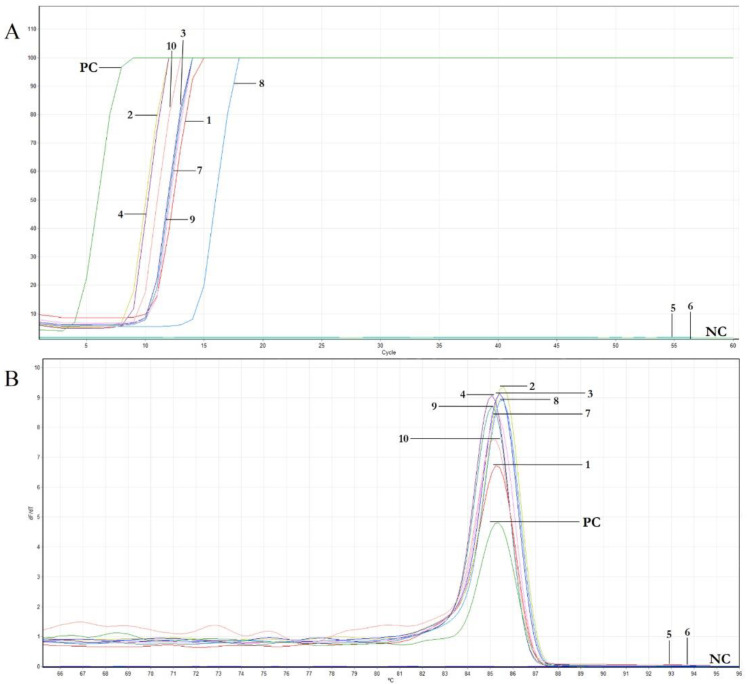
Results of the real time LAMP assay for the detection of OEGV. (**A**): Amplification curves of real-time LAMP assay; (**B**): Melting curves of the amplification curves previously obtained, including positive (PC) and negative control (NC).

To evaluate the performances of the primer set designed for the real time LAMP assay in the identification of the presence of OEGV in olive DNA extracts, the LAMP assay was conducted using a subset of the samples listed in Table 1, selecting them among those that resulted positive in end point PCR. In the assay, a sample that tested negative was also included (i.e., cv. Giarraffa), together with an appropriate negative control (NC). The assay was conducted at 65 °C. As reported in Table 3 and Figure 2A, the positive samples showed exponential trends between 3 to 13 min. The melting curves of the positive LAMP reactions all had the same peak temperature of approximately 85 °C (Figure 2B). As expected, no signal was obtained with the negative control and, according to the end point PCR results, the samples from cv. Giarraffa could not be amplified by LAMP, even at late reaction times.

### 3.4. Features of Real-Time LAMP Assay: Sensitivity and Comparison to Conventional PCR, Reaction Time and Specificity

**Figure 3:**
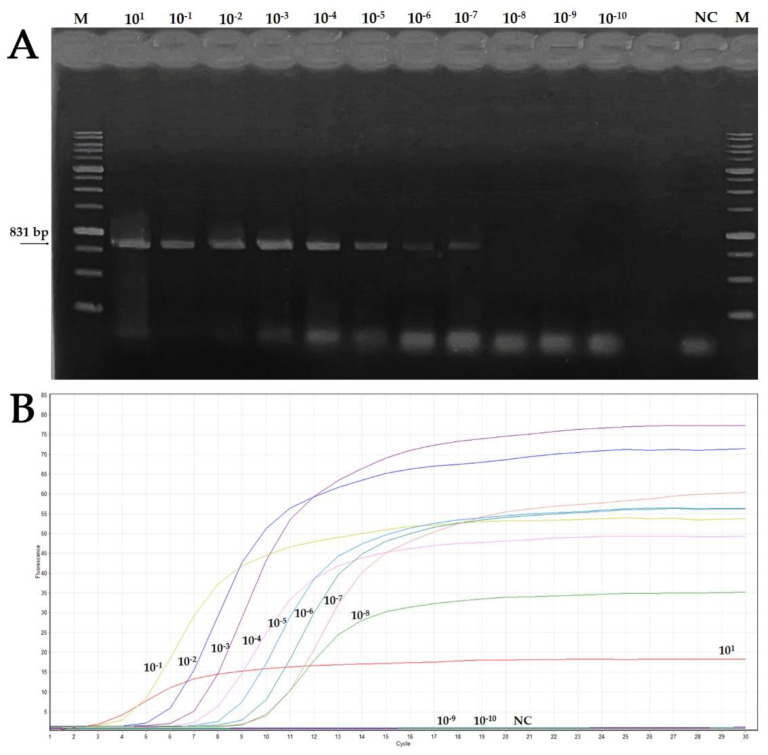
Sensitivity of the end point PCR (panel **A**) and real-time LAMP (panel **B**) for OEGV detection. The assay was conducted using 10-fold serial dilutions of pcr-DNA. Panel (**A**): Agarose gel electrophoresis of PCR products; M: 1 Kb ladder marker, NC: negative control. Panel (**B**). Fluorescence of the 10-fold serial dilutions analyzed. Fluorescence increased in positive sample curves (from 10^−1^ to 10^−8^) after 3 to 10 min.

To determine the sensitivity of the real-time LAMP assay compared to the end-point PCR and to evaluate the LAMP efficacy, a comparative experiment was conducted using as a template ten-fold serial dilutions of an amplicon obtained by end point PCR from an OEGV-positive sample (pcr-DNA), starting from a concentration of 80.9 ng/μL. As can be observed in Table 4 and Figure 3, DNA concentration up to ~80.9 × 10^−8^ ng/μL was detected with the LAMP assay, while the end point PCR positive signals were obtained with DNA concentration up to ~80.9 × 10^−7^ ng/μL, indicating that real time LAMP was about ten times more sensitive than conventional PCR.

Moreover, even considering the lowest detectable concentration of the pcr-DNA sample in real time LAMP (~80.9 × 10^−8^ ng/μL), the results clearly showed that the time required to carry out the experiment was less than 30 min.

In addition, to evaluate the specificity of the LAMP assay and to assess potential non-specific cross reactions with other geminiviruses present in the agricultural areas where olive crop samples were collected, we conducted a LAMP assay using the geminiviruses reported in paragraph 2.6 as a template. Results showed that no signals were obtained with any of the geminiviruses used as the outgroup, while the two OEGV-positive olive DNA samples used as controls reacted in real time LAMP with a time value of 10 min and a single peak at 85 °C in the melting curve. This allowed us to confirm the specificity of the assay and to exclude the cross-reactivity with unrelated geminiviruses previously isolated in Sicily.

### 3.5. Set up of a Rapid Sample Preparation Method Suitable for the Real-Time LAMP Assay

With the purpose of identify a method that allows a simple and inexpensive sample preparation useful for real time LAMP, samples prepared with the two different procedures were tested. For this, the ten samples previously analyzed by end-point PCR and by real time LAMP assay (Table 3) were considered. As reported in Table 5, all samples tested positive in the LAMP assay when extracted with either procedure. Specifically, samples extracted with the commercial kit showed a fluorescence increase ranging between 3–14 min, while the same samples prepared with the “membrane spot crude extract” method could be detected in 10–24 min. This is worthy of note, as it indicates that the rapid method allows for the detection of the presence of OEGV with a delay of only a few minutes compared to the corresponding extract obtained with the commercial kit. As expected, even with this rapid procedure, no reaction was obtained with the samples from cv. Giaraffa.

### 3.6. Spread of OEGV in Sicily

To investigate the spread of OEGV in different olive cultivars grown in Sicily, a new survey was conducted testing 70 samples, each consisting of eight different trees of the same cv. These samples were extracted with the rapid extraction protocol and tested in real-time LAMP, thus representing a total of 560 olive trees analyzed overall. This analysis showed that 30 out of the 70 cultivars (~43%) were positive for OEGV, indicating a relatively high incidence and prevalence of OEGV in the sampling locations and across cultivars (Table 6). When each of the eight plant samples present in the 30 positive batches were tested individually, the majority (235 out of 240 plants) resulted as being positive for OEGV, except the batch of cv. ‘Calatina’, where only three plants out of eight were positive (Table 6).

## 4. Discussion

The olive tree is affected by many potential pathogens, including viruses. Some of them are reported to be transmitted by different vectors [9,27,28,29], but the use of infected propagating material might represent the major, though not the only, means of virus spread [29,30,31,32,33]. The first report on a probable viral disease of olive goes back to 1938 [34] and, since then, several virus-like diseases and viruses have been reported over the years in different areas where olive cultivation plays a prominent role [14,35,36,37,38,39,40]. Some of these are agents of recognized diseases, others cause latent infections with still undetermined effects on the host [29]. The discovery of OEGV adds to the list of unclassified members of the family *Geminiviridae*, whose genome sequences diverge significantly from those of classified members [16]. The identification of this new virus was facilitated by HTS, a technique that allows for the discovery of new plant viruses, especially when symptoms are not evident, as is the case of OEGV. Besides Apulia, OEGV was recently reported in different areas where the olive cultivation is widespread [15,17,18].

To our knowledge, this study represents the first report of OEGV in Sicily. Since PCR-based methods can be affected by several inhibitors [41,42], such as phenols and polysaccharides [43,44] and require nucleic acid extraction methods [45], we aimed to develop a rapid detection method for OEGV based on LAMP. Indeed, this detection technique showed optimal characteristics, providing rapid, sensitive, specific, and easy detection of several pathogens even in the field, showing a reduced sensitivity to inhibitors [42,46]. The AV1 (CP) gene of OEGV was selected as the target region for primer design and the set of the six LAMP primers showed good specificity and stability for OEGV detection. The specificity is crucial to obtain correct discrimination of OEGV from other viruses belonging to the large *Geminiviridae* family, and a high sensitivity is relevant to minimize false negatives. A LAMP assay optimization performed using DNA extracted from OEGV-infected olive samples revealed that the time required to carry out the experiment was 30 min. The LAMP assay could detect the virus presence from infected samples in as little as 3–14 min. Interestingly, the real-time LAMP developed here has proven to have a 10-fold higher sensitivity compared to the end-point PCR for detection of OEGV. Moreover, in this study, the conventional extraction method using a commercial kit was compared with a “membrane spot crude extract” method; the data obtained from this comparison suggests that the LAMP-based detection method could be suitable for direct use in the field, confirming that ease of sample preparation is a crucial requirement for future application for on-site detection. Specifically, we demonstrated that the rapid sample preparation method allowed for the avoidance of DNA extraction and could be applied for future epidemiological studies, drastically reducing the cost of the analysis. Furthermore, this real-time LAMP technique, associated with other rapid extraction methods developed in other works [47], could be fine-tuned for an efficient and rapid in-field diagnosis.

The rapid extraction method definitely simplified the surveys of the OEGV spread in different cultivars in Sicily. The effectiveness of the techniques developed is essential to understand its spread and to refine effective methods of crop protections, in order to quickly diagnose the presence of a new pathogen in different areas [48,49,50].

Our survey revealed a considerable presence of the virus in the olive crops in Sicily, probably due to the inadvertent movement of clonally propagated infected but asymptomatic germplasms. Related to this, the development of diagnostic protocols for plant virus detection [51,52] and the epidemiological studies [53] of viral diseases are among the most important and useful steps towards the containment of new epidemics [54,55,56].

In conclusion, the real-time LAMP assay described in this work is a rapid, simple, specific and sensitive technique for detecting the presence of the recently described OEGV, allowing for the processing of a great number of samples at the same time, especially if associated with the “membrane spot crude extract” method. For this reason, we propose to adopt this method for routine tests in the laboratory and field conditions for a timely detection of OEGV. In particular, this method represents a potential tool for rapidly screening olive plant material useful for large surveys of the spread and pathogenicity of this virus, which to date remain uncertain. Interestingly, as recently reported by Ruiz-García and co-workers [18], the high level of sequence conservation encountered among all OEGV accession so far isolated requires a prompt investigation of the evolutionary and biological significance of this geminivirus in olive, opening new scenarios about its mechanisms of spread in the major olive-growing areas of the world.

## Figures and Tables

**Table 1:** Prevalence and cultivar distribution of OEGV analyzed by end-point PCR.

Cultivar	No. Positive/Tested Samples	Percentage of Positive Samples (%)
Cavalieri Standard	8/8	100
Cerasuola Nilo Paceco	8/8	100
Cerasuola Standard	8/8	100
Giarraffa	0/8	0
Nocellara del Belice Giafalione	8/8	100
Pizzutella	8/8	100
Salicina Vassallo	3/8	37.5
Uovo di piccione	1/8	12.5
Vaddara	0/8	0
Zaituna Florida	0/8	0
**Total**	**44/80**	**55**

**Table 2:** Primers used for OEGV detection by LAMP.

Primer Name	Sequence (5′-3′)	Amplicon Size (bp)
F3-OEGV	CGATACGAGACATACCCAG	209
B3-OEGV	TCCATGTTGATCATCCAAGT	
FIP-OEGV	CAGCCACTGCTTCATATTATGAACACGAATTGTGCTTAACGGTT	-
BIP-OEGV	GATGTGGCTCGTGTATGATAGACGTCTGGATCCCGACTTTCC	
LF-OEGV	GGCTTCGCTAGTCAACTTAACTG	-
LB-OEGV	TCCCGGTAATTCTAATCCCAGAG	

**Table 3:** Performance of the real time LAMP assay for the detection of OEGV in olive samples collected in Sicily.

Cultivar	No. of Different Samples Analyzed	ID Sample	Reaction Time (min)
Cavalieri Standard	2	1	10
		2	7
Cerasuola Standard	2	3	10
		4	7
Giarraffa	2	5	-
		6	-
Nocellara del Belice Giafalione	2	7	10
		8	13
Pizzutella	2	9	10
		10	9
Positive control	1	PC	3
Negative control	1	NC	-

**Table 4:** Comparison of the sensitivity of the real time LAMP and end-point PCR.

	Starting DNA Concentration (80.9 ng/μL)										
Assay	10^1^	10^−1^	10^−2^	10^−3^	10^−4^	10^−5^	10^−6^	10^−7^	10^−8^	10^−9^	10^−10^
End-point PCR	+	+	+	+	+	+	+	+	−	−	−
Real-time LAMP	+	+	+	+	+	+	+	+	+	−	−
Reaction Time (min)	3	4	5	6	7	8	9	10	10	−	−

**Table 5:** Comparison of two different sample preparation methods for the identification of the presence of OEGV in olive samples.

Cultivar	No. Samples Analyzed	Time Value		
		ID Sample	Total DNA Extraction by Commercial Kit (min)	Membrane Spot Crude Extract (min)
Cavalieri Standard	2	1	10	14
		2	7	12
Cerasuola Standard	2	3	10	16
		4	7	10
Giarraffa	2	5	-	-
		6	-	-
Nocellara del Belice Giafalione	2	7	10	15
		8	14	24
Pizzutella	2	9	10	16
		10	9	14
Positive control	1	PC	3	12
Negative control	1	NC	-	-

**Table 6:** Incidence of OEGV evaluated using a real-time LAMP assay on samples prepared with the “membrane spot crude extract” method.

Cultivar Analyzed	Real-Time LAMP	
	Cultivar Batch	Positive Plants/Tested Plants
Abunara	+	8/8
Aitana	−	NT
Arbequina	+	8/8
Bariddara	+	8/8
Biancolilla Caltabellotta	−	NT
Biancolilla Caltabellotta TA PC	+	8/8
Biancolilla Iacapa	−	NT
Biancolilla Napoletana	−	NT
Biancolilla Pantelleria	−	NT
Biancolilla Schimmenti	−	NT
Biancolilla Siracusana	−	NT
Biancuzza	−	NT
Bottone di Gallo Vassallo	−	NT
Brandofino	−	NT
Calamignara	−	NT
Calatina	+	3/8
Carasuola Cappuccia	+	8/8
Castricianella Rapparina	+	8/8
Cavalieri Standard	+	8/8
Cerasuola 1 Clone 2	+	8/8
Cerasuola Nilo Paceco	+	8/8
Cerasuola Standard	+	8/8
Conservolia	−	NT
Crastu Collesano	−	NT
Galatina	−	NT
Giarraffa	−	NT
Gordales	−	NT
Iacona	+	8/8
Indemoniata	−	NT
Koroneiki	+	8/8
Leucocarpa	−	NT
Lunga di Vassallo	+	8/8
Manzanilla	−	NT
Minna di Vacca	−	NT
Minuta	+	8/8
Monaca	+	8/8
Moresca	−	NT
Murtiddara Vassallo	+	8/8
Nasitana	+	8/8
Nocellara del Belice Giafalione	+	8/8
Nocellara del Belice Clone 1	−	NT
Nocellara del Belice Clone 7	−	NT
Nocellara del Belice Mazara del Vallo	−	NT
Nocellara del Belice Standard	−	NT
Nocellara Etnea	−	NT
Nocellara Messinese Ricciardi	−	NT
Nocellara Messinese Romana	−	NT
Ogliara Maltese	−	NT
Oliva Longa	−	NT
Olivo di Mandanici	+	8/8
Olivo di Monaci	+	8/8
Opera Pia	+	8/8
Passalunara di Lascari	−	NT
Picholine	−	NT
Piricuddara	+	8/8
Pizzo di Corvo	−	NT
Pizzuta d’Olio	+	8/8
Pizzutella	+	8/8
Salicina Vassallo	−	NT
Tonda Iblea	−	NT
Tortella Motticiana	−	NT
Tunnilidda	−	NT
Uovo di Piccione	−	NT
Vaddara	−	NT
Vaddarica	+	8/8
Verdella	+	8/8
Verdella Frutto Grosso	+	8/8
Verdello	+	8/8
Vetrana	+	8/8
Zaituna Floridia	−	NT

**Note**: +: positive sample; −: negative sample; NT: Not Tested.

## Data Availability

Not applicable.
